# Analysis of the Usability of Iron Ore Ultra-Fines for Hydrogen-Based Fluidized Bed Direct Reduction—A Review

**DOI:** 10.3390/ma15072687

**Published:** 2022-04-06

**Authors:** Thomas Wolfinger, Daniel Spreitzer, Johannes Schenk

**Affiliations:** 1 K1-MET GmbH, Stahlstraße 14, 4020 Linz, Austria; johannes.schenk@unileoben.ac.at; 2 Primetals Technologies Austria GmbH, Turmstraße 44, 4020 Linz, Austria; daniel.spreitzer@primetals.com; 3 Ferrous Metallurgy, Montanuniversitaet Leoben, Franz-Josef-Straße 18, 8700 Leoben, Austria

**Keywords:** direct reduction, hydrogen, fluidized bed, iron ore ultra-fines, sticking

## Abstract

This review focuses on the usability of iron ore ultra-fines for hydrogen-based direct reduction. Such technology is driven by the need to lower CO_2_ emissions and energy consumption for the iron and steel industry. In addition, low operational and capital expenditures and a high oxide yield because of the direct use of ultra-fines can be highlighted. The classification of powders for a fluidized bed are reviewed. Fluid dynamics, such as minimum fluidization velocity, entrainment velocity and fluidized state diagrams are summarized and discussed regarding the processing of iron ore ultra-fines in a fluidized bed. The influence of the reduction process, especially the agglomeration phenomenon sticking, is evaluated. Thus, the sticking determining factors and the solutions to avoid sticking are reviewed and discussed. The essential theoretical considerations and process-relevant issues are provided for the usability of iron ore ultra-fines for hydrogen-based fluidized bed direct reduction.

## 1. Introduction

The iron and steel industry accounts for one-third of the global industrial CO_2_ emissions. Measures and new technologies have to be implemented in the coming decades to achieve the ambitious goals for a carbon-free economy, e.g., by 2050 for the European Union [1,2,3]. The dimensions of these forthcoming measures become tangible by looking at the global iron ore and crude steel production, summarized in Table 1. In 2020, the world’s crude steel production amounted to 1.88 billion tons, whereby nearly three quarters was produced via the basic oxygen furnace (BOF) [4]. The primary iron input material for the BOF is hot metal and 20 to 30% scrap [5]. For the production of crude steel via the electric arc furnace (EAF), mainly scrap is used, although direct reduced iron (DRI), hot briquetted iron (HBI) and pig iron (i.e., solidified hot metal) can be charged if scrap is not available in the required quality and/or quantity. The primary CO_2_ emissions contributor of the iron and steel industry is the blast furnace–basic oxygen furnace route (BF–BOF), which emits appr. 1.7 to 1.9 tons CO_2_ per ton of crude steel [6,7,8]. Besides the blast furnace, smelting reduction (SR) and direct reduction (DR) technologies produce virgin iron materials, which are hot metal, pig iron and DRI/HBI, from iron ore. These three technologies are summarized within the family of ironmaking technologies. In contrast, the EAF route mainly uses recycled material as feed material, but also DRI/HBI or pig iron. A promising route to reduce specific CO_2_ emissions is hydrogen-based direct reduction in combination with a melting furnace [3,6,8,9,10]. Compared to the BF–BOF route, CO_2_ emissions can be halved using natural gas as a basis for the reduction process [7]. A further reduction towards 0.1 to 0.25 tons CO_2_ per ton of crude steel is theoretically achievable by using hydrogen generated from electrolysis with renewable energies [6].

**Figure 1:**
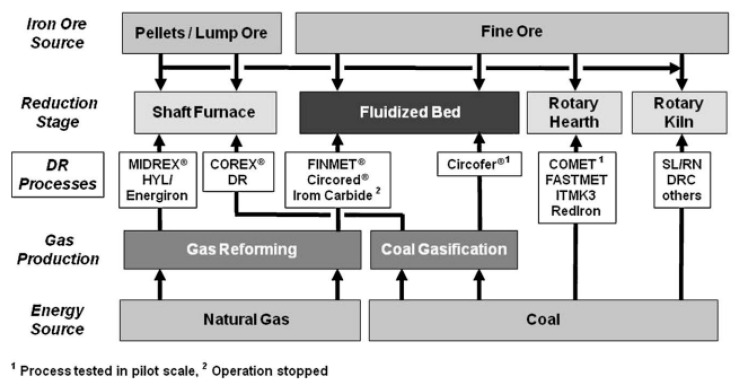
Classification of direct reduction processes [5].

In addition to being the main driver of lowering CO_2_ emissions, new ironmaking technologies need to be able to process any type of iron ore directly, preferably in the form of ultra-fines, owing to the steadily increasing amount of ultra-fines resulting from the intensified beneficiation of low-grade ore deposits [13]. Directly using iron ore ultra-fines further decreases energy consumption and CO_2_ emissions due to the omission of the agglomeration process (e.g., pelletizing) [3,8,14]. Based on these technological circumstances, the fluidized bed technology using hydrogen is of interest [5,15]. Schenk classified direct reduction processes according to the iron ore and energy source, gas production and reactor system for the reduction stage, given in Figure 1 [5].

The Finmet^®^ and Circored^®^ processes are identified as direct reduction technologies that directly use a hydrogen-rich reducing gas and iron ore fines. The feed material for the Finmet^®^ process is sinter feed ore, meaning a particle size distribution mainly between 0.05 and 8 mm, while the feed material for the Circored^®^ process is between 0.1 and 2.0 mm, meaning crushing of oversized sinter feed ore and microgranulation of undersized pellet feed ore [5,15,16,17,18]. The newly developed HYFOR^®^ process shows similarities to Finmet^®^ and Circored^®^ by means of a direct reduction technology using a hydrogen-rich gas or even 100% hydrogen as the reducing gas and iron ore fines directly [19]. However, the feed material is in the form of ultra-fines, such as pellet feed ore, i.e., a particle size distribution of <150 µm. The advantages of such a process are summarized as follows [19]:

- Low CO_2_ footprint using hydrogen-rich gas or even 100% hydrogen;
- Low operational and capital expenditures as well as overall energy consumption per ton of product due to the omission of the agglomeration step (i.e., pelletizing);
- High iron oxide yield due to recycling of oxide dust and usage of undersize fraction as feed material.

To achieve these advantages, technological challenges need to be solved in the HYFOR^®^ process development. The main issue for a direct reduction process based on fluidized bed technology is to keep the fluidization stable throughout the transformation of the iron oxide phases of the ore to metallic iron. Several authors have reported fluidization problems caused by changing surface morphology of the iron ore particles during reduction and due to sticking [20,21,22,23,24,25,26,27,28,29,30,31,32]. In addition, forming a dense iron layer around the particles prolongs the reduction [21,29,33,34]. Hence, for hydrogen-based reduction of iron ore ultra-fines in a fluidized bed, it is critical to focus on the fluid dynamics of the fluidization and the reduction in terms of morphological evolution of the material.

The following sections examine theoretical considerations and influences for processing iron ore ultra-fines in a hydrogen-based fluidized bed reactor. This includes theoretical considerations and the effect of hydrogen-based reduction for the usage of iron ore ultra-fines in a fluidized bed. The classification of powders for a fluidized bed, the minimum fluidization velocity, the entrainment velocity and fluidized state diagrams are summarized and discussed. For the effect of the process conditions, the essential issue for a stable fluidization—the agglomeration phenomenon sticking—is reviewed and evaluated. This includes the different types of sticking, the influencing factors and solutions to avoid sticking.

## 2. Classification of Powders for Fluidized Bed

**Figure 2:**
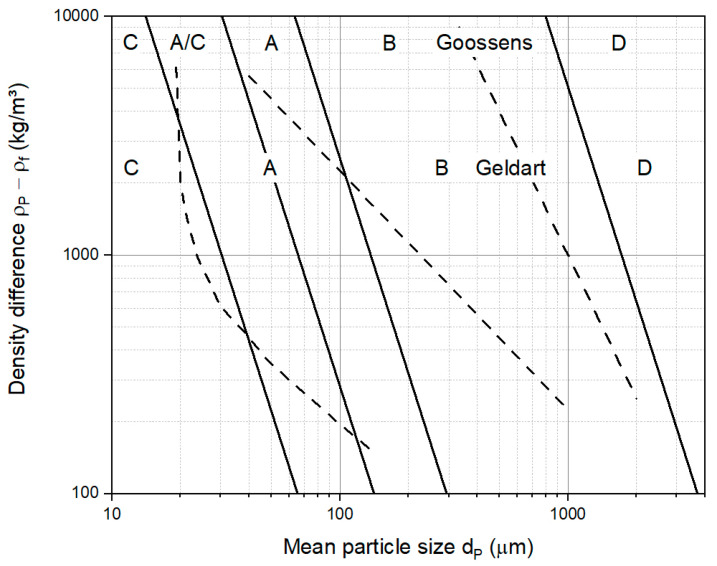
General classification diagram for fluidized particles in ambient conditions and air, including boundaries based on Geldart [35], dashed lines and Goossens [36], solid lines.

A common way to determine the fluidization behavior of a material in a fluidized bed reactor is by using Geldart’s generalized map for powder classification [35]. This map is given as a plot of the density difference between the particles and the fluidizing medium $\left(\rho_{p}-{\;\rho }_{f}\right)$ and the mean particle size $d_{p}$, shown in Figure 2. The solid materials are divided into the following four groups, according to their fluidization behavior:

- Group A: Fine and aeratable solids which show a significant bed expansion above the minimum fluidization velocity and have a minimum bubbling velocity, and thus good fluidization quality;
- Group B: Intermediate-sized particles which hardly show bed expansion; bubbles start to appear when fluidization is obtained;
- Group C: Very fine and cohesive materials which show the formation of channels and agglomerates rather than a fluidization behavior, and are therefore difficult to fluidize at all;
- Group D: Coarse particles which show bubbles rising and coalescence, and form vertical channels rather than a fluidization behavior.

In contrast to the experimentally found boundaries used for the classification form Geldart, Goossens suggested the Archimedes number to define the boundaries between the groups [36]. The Archimedes number, Ar, is defined as follows:

(1) $$\mathrm{Ar}=\frac{d_{p}^{3}{\;*\;\rho }_{f}\;*\;\left(\rho_{p}-{\;\rho }_{f}\right)*\;g}{\eta_{f}^{2}}$$

where $d_{p}$ is the mean particle size, $\rho_{p}$ is the particle density, $\rho_{f}$ is the density of the fluidizing medium, g is the gravity (9.81 m/s²) and $\eta_{f}$ is the dynamic gas viscosity.

These boundaries are derived from the transition points between viscous (laminar) and kinetic (turbulent) flow effects for the situation of entrainment or minimum fluidization and the corresponding correlations between the Reynolds number, Re, and the Archimedes number. The Reynolds number, in this case for particles, is defined as follows:

(2) Re=dp ∗ ρf ∗ uslipηf

where $u_{\mathrm{slip}}$ is the slip velocity, given by the superficial gas velocity in the reactor, u, divided by the void fraction of the bed material in fluidized state, $\varepsilon_{f}$, as follows:

(3) $$u_{\mathrm{slip}}=\frac{u}{\varepsilon_{f}}$$

(4) $$u=\frac{\dot{V}}{A}$$

(5) $$\varepsilon_{f}=1\;-\frac{\rho_{\mathrm{bed}}}{\rho_{p}}$$

where $\dot{V}$ is the gas flow through the reactor, A is the cross-sectional area of the reactor and $\rho_{\mathrm{bed}}$ is the bed density in a fluidized state. What is similar to the void fraction of the bed material, $\varepsilon_{f}$, is the void fraction of the bulk material, ε, calculated using the bulk density instead of the bed density. For the calculation of the boundaries between the groups according to Goossens, gas properties for air at ambient conditions were used, resulting in Archimedes numbers of 0.97, 9.8, 88.5 and 176,900 for the boundaries between the groups C, A/C, A, B and D, respectively, illustrated in Figure 2 as solid lines. The experimentally found boundaries for Geldart are shown in Figure 2 as dashed lines.

## 3. Theoretical Considerations for the Processing of Iron Ore Ultra-Fines in a Fluidized Bed

The theoretical background of the fluidized bed technology with a focus on the usage of iron ore ultra-fines is reviewed in this section. The minimum fluidization and entrainment velocity for ultra-fine feed material are analyzed and fluidized state diagrams to describe the fluidization are discussed.

### 3.1. Prediction of the Minimum Fluidization Velocity

The lower limit for a fluidized bed process is defined by the minimum fluidization velocity, $u_{\mathrm{mf}}$. This velocity is characteristic for bulk material and can be determined for the force equilibrium of the pressure-loss force caused by the gas flow through the packed bed and the weight of the bulk material. A description of the pressure loss through the packed bed is given by the equation proposed by Ergun, as follows [37]:

(6) $$\frac{\Delta P}{H}{=K}_{l}\;*\;\frac{{\left(1\;-\;\varepsilon \right)}^{2}{\;*\;\eta }_{f}\;*\;u}{\varepsilon^{3}\;*\;\phi^{2}{\;*\;d}_{p}{}^{2}}{+K}_{t}\;*\;\frac{\left(1\;-\;\varepsilon \right){\;*\;\rho }_{f}{\;*\;u}^{2}}{\varepsilon^{3}\;*\;\phi {\;*\;d}_{p}}$$

where ΔP is the pressure drop caused by a gas flow through a packed bed, H is the height of the packed bed and ϕ is the sphericity of the particles. The pressure drop is a function of viscous and inertial effects, which are given on the right-hand side of the equation, respectively. The constants $K_{l}$ and $K_{t}$ are for spherical particles 150 and 1.75, respectively, but have to be determined experimentally, especially for non-spherical particles [38,39].

At minimum fluidization, the pressure loss across the bed must be balanced by the effective weight of the bed:

(7) $$\frac{\Delta P}{H}=\left(1\;-\;\varepsilon \right)\;*\;\left(\rho_{p}-{\;\rho }_{f}\right)\;*\;g$$

At the point of incipient fluidization, Equations (6) and (7) can be combined. Multiplying both sides by the term $\frac{d_{p}{}^{3}{\;*\;\rho }_{f}}{\left(1\;-\;\varepsilon \right){\;*\;\eta }_{f}{}^{2}}$ leads to the following equations, whereby Equation (9) is rewritten using Re and Ar:

(8) $$\frac{d_{p}{}^{3}{\;*\;\rho }_{f}\;*\;\left(\rho_{p}-{\;\rho }_{f}\right)\;*\;g}{\eta_{f}{}^{2}}{=K}_{l}\;*\;\frac{(1\;-{\;\varepsilon }_{\mathrm{mf}})}{\varepsilon_{\mathrm{mf}}{}^{3}\;*\;\phi^{2}}\;*\;\left(\frac{d_{p}{\;*\;\rho }_{f}{\;*\;u}_{\mathrm{mf}}}{\eta_{f}}\right){+K}_{t}\;*\;\frac{1}{\varepsilon_{\mathrm{mf}}{}^{3}\;*\;\phi }\;*\;{\left(\frac{d_{p}{\;*\;\rho }_{f}{\;*\;u}_{\mathrm{mf}}}{\eta_{f}}\right)}^{2}$$

(9) Ar=Kl ∗ (1 − εmf)εmf3 ∗ ϕ2 ∗ Remf+Kt ∗ 1εmf3 ∗ ϕ ∗ Remf2

Rearranging Equation (8) leads to $u_{\mathrm{mf}}$ as follows:

(10) $$u_{\mathrm{mf}}\;=\;\frac{-\frac{K_{l}\;*\;\left(1\;-{\;\varepsilon }_{\mathrm{mf}}\right)}{\varepsilon_{\mathrm{mf}}^{3}\;*\;\phi^{2}}*\frac{d_{p\;}{\;*\;\rho }_{f}}{\eta_{f}}+\sqrt{{\left(\frac{K_{l}\;*\;\left(1\;-{\;\varepsilon }_{\mathrm{mf}}\right)}{\varepsilon_{\mathrm{mf}}^{3}\;*\;\phi^{2}}\;*\;\frac{d_{p}{\;*\;\rho }_{f}}{\eta_{f}}\right)}^{2}\;-\;4\;*\;\frac{K_{t}}{\varepsilon_{\mathrm{mf}}^{3}\;*\;\phi }\;*{\left(\frac{d_{p}{\;*\;\rho }_{f}}{\eta_{f}}\right)}^{2}*\;\left(-\frac{d_{p}^{3}{\;*\;\rho }_{f}\;*\;\left(\rho_{p}\;-{\;\rho }_{f}\right)\;*\;g}{\eta_{f}{}^{2}}\right)}}{2\;*\;\frac{K_{t}}{\varepsilon_{\mathrm{mf}}^{3}\;*\;\phi }*{\left(\frac{d_{p}{\;*\;\rho }_{f}}{\eta_{f}}\right)}^{2}}$$

In the case of iron ore ultra-fines, the bulk material consists of small particles (i.e., <150 µm) only; the viscous term is dominant and the inertial term can be neglected. Thus, Equation (10) can be rewritten:

(11) $$u_{\mathrm{mf}}=\frac{1}{K_{l}}\;*\;\frac{\varepsilon_{\mathrm{mf}}{}^{3}\;*\;\phi^{2}}{(1\;-{\;\varepsilon }_{\mathrm{mf}})}\;*\;\frac{\left(\rho_{p}-{\;\rho }_{f}\right){\;*\;g\;*\;d}_{p}{}^{2}}{\eta_{f}}$$

Several authors have applied approximations and simplifications for the terms $\frac{(1\;-{\;\varepsilon }_{\mathrm{mf}})}{\varepsilon_{\mathrm{mf}}{}^{3}\;*\;\phi^{2}}$ and $\frac{1}{\varepsilon_{\mathrm{mf}}{}^{3}\;*\;\phi }$ to cope with the lack and difficulty of measuring these parameters. The most common simplification for calculating $u_{\mathrm{mf}}$ is the one proposed by Wen and Yu, known as the Wen–Yu equation [40]:

(12) $$u_{\mathrm{mf}}=\frac{1}{1650}\;*\;\frac{\left(\rho_{p}-{\;\rho }_{f}\right){\;*\;g\;*\;d}_{p}{}^{2}}{\eta_{f}}$$

where the terms $\frac{(1\;-{\;\varepsilon }_{\mathrm{mf}})}{\varepsilon_{\mathrm{mf}}{}^{3}\;*\;\phi^{2}}$ and $\frac{1}{\varepsilon_{\mathrm{mf}}{}^{3}\;*\;\phi }$ are approximated by (11) and (14), respectively.

### 3.2. Prediction of the Entrainment Velocity

For the prediction of the upper limit of the operating field of a fluidized bed process, the entrainment velocity, $u_{t}$, is calculated by balancing the acting forces on a particle in fluidized state as follows:

(13) $$F_{G}-{\;F}_{B}-{\;F}_{D}=0$$

where $F_{G}$ is the gravitational, $F_{B}$ is the buoyancy and $F_{D}$ is the drag force due to the gas flow. Inserting the mean particle diameter as the representative length and the drag coefficient for a single particle, $C_{D}$, the equation can be written:

(14) $$\frac{{\pi \;*\;d}_{p}{}^{3}}{6}\;*\;\left(\rho_{p}-{\;\rho }_{f}\right)\;*\;g\;-{\;C}_{D}\;*\;\frac{{\pi \;*\;d}_{p}{}^{2}}{4}\;*\;\frac{\rho_{f}}{2}{\;*\;u}_{t}{}^{2}=0$$

**Figure 3:**
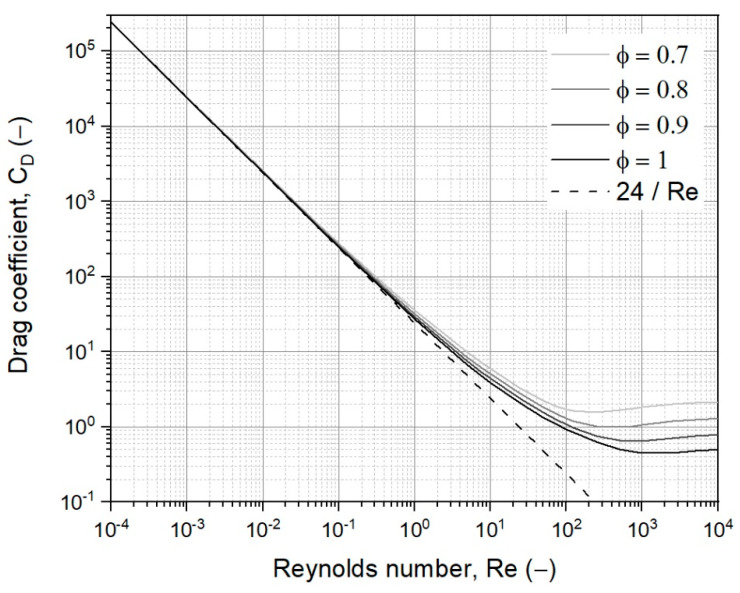
Drag coefficient for a single free-falling particle as a function of the Reynolds number based on Haider and Levenspiel [41], solid lines, and the simplification for laminar flow, the dashed line.

The drag coefficient for a single particle is a function of the Reynolds number, illustrated as solid lines in Figure 3, and can be calculated according to the formula established by Haider and Levenspiel for different particle sphericities as follows [41]:

(15) CD=24Re ∗ [1+(8.1716 ∗ e−4.0655 ∗ ϕ) ∗ Re0.0964+0.5565 ∗ ϕ]+73.9 ∗ (e−5.0748 ∗ ϕ) ∗ ReRe+5.378 ∗ e6.2122 ∗ ϕ

By inserting the Archimedes number and the rearranged Reynolds number for $u_{t}$ in Equation (14), the following equation is obtained:

(16) CD ∗ Re2=43 ∗ Ar

For the case of a bulk material consisting of small particles only (laminar flow for Re ≤ 0.5), the drag coefficient is reversed proportional to the Reynolds number, as CD=24/Re, shown in Figure 3 as a dashed line. Thus, the resulting equation for the entrainment velocity, also known as Stoke’s equation, is given as:

(17) $$u_{t}=\frac{1}{18}\;*\;\frac{\left(\rho_{p}-{\;\rho }_{f}\right){\;*\;g\;*\;d}_{p}{}^{2}}{\eta_{f}}$$

### 3.3. Fluidized State Diagrams to Describe the Fluidization

**Figure 4:**
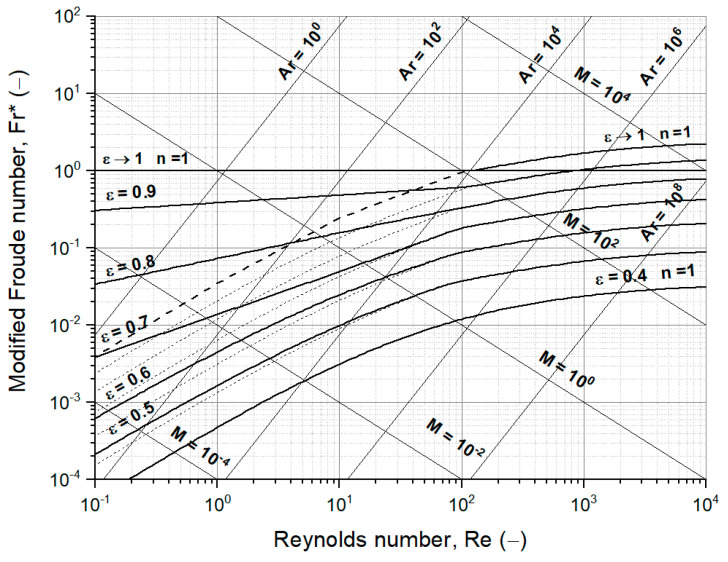
Fluidized state diagram following Reh with the extended area of fluidized beds for small particles from homogenous fluidization [43], dashed lines, to heterogenous fluidization, solid lines.

For the fluidization of iron ore ultra-fines, the case of a single particle is only valid when the particle is carried out of the fluidized bed. Otherwise, mutual interactions of the particles have to be considered. This is achieved with the equation proposed by Richardson and Zaki for homogenous fluidization [42]:

(18) CD(Re,ε)=CDεα

where by α is an index and depends on the Reynolds number for particles. With this equation, the whole process area from the packed bed to homogenous fluidization of the bulk material up to the entrainment velocity of a single particle can be described. In a more technical description, using the load factor, n, which is the balance of upwards to downwards acceleration: from packed bed n ≤ 1, to fluidized bed n = 1, and to pneumatic conveying n ≥ 1. This relationship is only valid for homogenous fluidization (particulate fluidization) and the assumed void fraction for the packed bed, e.g., 0.4 for mono-disperse spherical particle systems. For heterogenous fluidized beds (aggregative fluidization), which are most of the gas–solid systems, the drag coefficient is assumed to be towards the value of one with an increasing void fraction. A value of one for the drag coefficient means that only the dynamic pressure without the resistance increasing influence of friction and without the resistance decreasing influence of vortex shedding is acting on the particles. As a result, an extension of the boundary between the fluidized bed and pneumatic conveying, ε → 1 and n = 1, for small particles for heterogenous fluidization is drawn. Considering these assumptions, one can draw the well-known fluidized state diagram following Reh in Figure 4, using the already introduced dimensionless numbers Reynolds number Re, Archimedes number Ar and the following two-dimensionless numbers: the modified Froude number ${\mathrm{Fr}}^{*}$ and Liatschenko number M [43]:

(19) Fr*=34 ∗ uslip2g ∗ dp ∗ ρfρp− ρf=34 ∗ Re2Ar=nCD(Re,ε)

(20) $$M=\frac{u_{\mathrm{slip}}^{3}{\;*\;\rho }_{f}^{2}}{{g\;*\;\eta }_{f}\;*\;\left(\rho_{p}-{\;\rho }_{f}\right)}$$

Besides Reh’s fluidized state diagram, similar diagrams to characterize the fluidization are given by the fluidization regime map of Grace or by the flow regime diagram of Rabinovich and Kalman or Shaul et al. [44,45,46,47]. Compared to the fluidized state diagram following Reh, only the area for homogenous fluidization is drawn, which means no considerations for the heterogenous fluidization are taken into account. However, some boundaries for different fluidization regimes are drawn based on empirically determined equations. Another difference is that the lower limit for the fluidized bed area is defined by the minimum fluidization velocity but based on simplified equations and thus only limited to iron ore ultra-fines. Shaul et al. also considered the bed height to diameter ratio for the flow regime diagram [45,46]. In addition, they also established a block-flow diagram to determine regimes for fluidized beds regarding the defined groups of Geldart, but without Group C and including the bed height to diameter ratio [45].

## 4. Influence of the Reduction Process on the Fluidization Behavior

The operating temperature of a reduction process for iron ore is well above ambient, and has different gas mixtures and elevated pressures. Several authors have evaluated diverse affecting variables accounting for particle and gas properties and process conditions for fluidized-bed operation [26,43,44,45,48,49]. In principle, three aspects have to be addressed, namely the thermodynamic aspect, the kinetic aspect and the need for stable fluidization to ensure an intense contact between the gas and solid. From the point of view of a plant operator of a hydrogen-based fluidized bed direct reduction plant, the following four conditions of the process need to be balanced to comply with the three aspects [5,26]:

- Feed material: as fine as possible to avoid kinetic limitations, but as coarse as possible to enable high gas-flow rates;
- Bed height: as low as possible to minimize heat losses (reactor size), but as high as possible to avoid kinetic limitations and fluidization difficulties;
- Reduction temperature: as low as possible to avoid thermally induced agglomeration, but as high as possible to avoid kinetic limitations and increase the gas utilization;
- Gas-flow rate: as low as possible to minimize entrainment, but as high as possible to maximize the supply of reactant and heat transfer rates and to avoid defluidization.

Focusing on the processing of iron ore ultra-fines, the feed material is chosen to be as fine as possible. The height of the fluidized bed can be estimated with the established block flow diagram of Shaul et al. [46], although laboratory tests to define the optimal height of the fluidized bed are not compensated. Process conditions such as reduction temperature and gas flow rate affect the reduction regarding fluidization behavior and thermodynamic and kinetic aspects. Nevertheless, several authors have identified the agglomeration phenomenon sticking as the primary issue for stable fluidization behavior, which is discussed in the next section [20,21,22,23,24,25,26,27,28,29,30,31,32].

### 4.1. The Sticking Phenomenon

Besides the advantages of fluidized bed direct reduction, the essential issue is to ensure stable fluidization throughout the whole process. Thus, the crucial problem is the sticking phenomenon, which leads to the agglomeration of particles and causes defluidization [22,23,26,27,29,50,51,52,53,54,55,56,57,58,59,60,61]. For iron oxide reduction, three different types are identified [23,29,52,61]:

- Type 1 sticking—the bonding effect of generated iron whiskers;
- Type 2 sticking—the bonding effect of generated new metallic iron;
- Type 3 sticking—the bonding effect due to low melting eutectica.

In the first type of sticking, the generated iron whiskers are fibrous metallic iron precipitation. Interlocking of these whiskers from particles causes the formation of agglomerates [28,52,61,62]. The conditions are given for the reaction-controlled situation when the generation rate of iron ions on the surface of the particles is much slower than the solid-state diffusion rate [23,34,63,64]. The entire particle is like a reservoir for storing iron ions until the critical lowest nucleation free energy at the surface is reached. At this point, the iron nuclei form and with the continuous supplement of iron ions, the whiskers grow away from the surface. Gong et al. explained the growth of iron whisker via vacancy defects on the surface of the iron whisker, which favors the diffusion of iron atoms und thus the sticking of the iron particles [28]. Gudenau et al. and Du et al. claimed that this type is not relevant for iron oxide reduction with hydrogen [23,58]. In contrast, Moujahid and Rist as well as Gransden and Sheasby reported the formation of iron whiskers for hydrogen-based reduction [34,51]. Hayashi and Iguchi found that the formation of iron whiskers during reduction with hydrogen can be suppressed by additional hydrogen sulfide in the gas [22,65]. The practical usage of this action is, however, questionable.

The second type of sticking results from the increased adhesion and friction among particles caused by the high surface energy and viscosity of the highly active new metallic iron [55,61,62,66]. Tardos et al. define the adhesive properties for a softened material with the surface viscosity of the particle, assuming its surface behaves as a Newtonian fluid, so surface deformation occurs under a given shear field [67]. Authors have also found that the solid-state diffusion of the freshly formed metallic iron results in interconnected solid bridges between the particles [56,66,68]. Consequently, the particles tend to agglomerate, especially at high reduction rates and temperatures. This type of sticking dominates for the reduction with hydrogen.

The third type of sticking can occur at a temperature above 1123 K because of the generation of low melting eutectica between wüstite and gangue components of the iron ore [29,52,69]. As a result, the particles become soft and tend to bond.

In addition to the three types of sticking, a fourth type is mentioned by some authors. This type accounts for van der Waals forces and magnetic attraction and is therefore only relevant when the particle size is below the micron level [29,61].

### 4.2. Influencing Factors of the Sticking Phenomenon

To classify the influencing factors on the sticking phenomenon, Langston and Stephens proposed a concept for the tendency of particles to stick together when they collide with each other [26]. They claimed that the tendency of sticking, S, is proportional to the contact area between colliding particles, A; and their adhesive properties, B; and inversely proportional to the momentum of the particles, C, as follows:

(21) $$S=f\;\left(\frac{A\;*\;B}{C}\right)$$

Based on this concept, several authors have identified the following influencing factors on the sticking phenomenon for the fluidized bed direct reduction [26,29,56,58].

- Reduction temperature;
- Reducing gas composition and gas flow rate;
- Metallization degree/reduction degree;
- Reduction time;
- Iron ore characteristics and particle size distribution.

The most significant process parameter for fluidized bed direct reduction to avoid defluidization is the reduction temperature. The tendency of sticking is affected by the temperature in two ways. First, a joint influence with the gas composition determines the metallic iron morphology [34,58,61,63]. Second, the higher the temperature, the higher the adhesive forces of metallic particles, especially for new highly active iron [27,53,54,70,71,72,73].

The second most significant process parameter is the reducing gas through composition and flow rate. First, as mentioned, a joint influence with the reduction temperature determines the metallic iron morphology. Second, as a significant influence of the reduction time [29,74,75]. Third, as the driving force for the momentum of the particles due to the acting drag force of the gas [53,54,76,77]. Thus, the greater the flow rate, the greater the drag force and momentum, the greater the agitation of the fluidizing bed, the greater the intensity of the collision among the particles and the shorter the contact time [29,50,78]. Reviewing the above-mentioned points with type 1 and type 2 sticking, sticking occurs after a given time when the first metallic iron has emerged. Several authors have reported a direct correlation between the tendency of sticking and both the metallization ratio and the reduction time [24,27,50,54,59,78,79].

Besides the process conditions, the characteristics of the feed material, including the pre-processing steps of the iron ore such as beneficiation, affect the sticking phenomenon in three ways. First, the higher the ore grade, the higher the sticking tendency, because lesser gangue components inhibit sticking to the metallic iron surface [29,50]. In the case of low-grade ore in combination with temperatures above 1123 K, type 3 sticking must be considered [29,52,69]. Second, the smaller the particles, the higher the contact area between colliding particles and the possibility for collisions and the smaller the momentum imparted, and thus, the higher the sticking tendency [50,53,56,61,80,81]. In addition, Zhong et al. found that a broad particle size distribution compared to a narrow particle size distribution shows a higher tendency towards sticking at elevated temperatures due to a lower sintering temperature [72]. Third, the shape of the particles because the higher the angularity, the higher the tendency of sticking [22,56,82].

Derived from the influencing factors, the process is mainly driven by the gas velocity, deduced from the gas flow rate and the reduction temperature. As an example, a classification accounting for reducing gas mixtures, gas velocities and temperatures was established in the form of a regime diagram for fluidization behavior by He et al. and Lei et al. [24,73]. In addition to the good applicability of such a regime diagram, the validity is restricted to the tested feed material and reducing gas.

Although, the reasons for the different sticking types and the influencing factors can be only explained to a limited extend as tendencies, the focus has to lay on solutions to avoid the sticking phenomenon.

### 4.3. Solutions to Avoid the Sticking Phenomenon

The avoidance of sticking is crucial for a stable fluidization operation. Over the past few decades, several methods for suppressing the sticking phenomenon have been topics of scientific and industrial research and development work. Derived from the definition of the different types and the influencing factors, applicable methods for hydrogen-based fluidized bed direct reduction are as follows:

- Increasing the gas flow rate;
- Adapting the form of fluidization;
- Lowering the reduction temperature;
- Controlling the metallic iron morphology;
- Coating treatment.

For the sake of completeness, other methods to avoid sticking in fluidized bed reduction have been reported, but these solutions are neither feasible for hydrogen-based direct reduction—such as carbon decomposition on the particle surface via gas phase or isolation treatment using coal-ore mixtures—nor industrially practicable, such as using acoustic or mechanical vibrators [29,53,61,83,84,85,86,87,88,89].

The gas flow rate is the driving force for the momentum of the particles. Consequently, increasing the gas flow rate increases the intensity of the collision among the particles and shortens the contact time, resulting in a lower sticking tendency. The increasing momentum of the particles by the gas flow can change the fluidization regime [29]. Many authors have found that the pulsation of the inlet flow improves the fluidization [90,91,92]. He et al. reported that an upward expansion of the fluidized bed improves the fluidization of the bubbling fluidized bed and partly reduces the sticking tendency [24]. In the case of lower reduction temperatures, as the most significant factor in suppressing the sticking problem, two aspects have to be considered. First, the lower the temperature, the lower the viscosity of the metallic surface of the particles. Second, in conjunction with controlling the metallic iron morphology, an upper limit for the temperature can be identified. Compared with the thermodynamically favored reduction rate for higher temperatures, an upper limit of the temperature is defined by the solid-state diffusion-controlled kinetic situation [34,63,64]. This situation prevails when the solid-state diffusion of the newly formed iron atoms is much slower than the generating rate of iron atoms. As a result, an accumulation of iron atoms at the surface of the particles occurs, leading to a dense iron shell around the particles, hindering the ongoing reduction due to solid-state diffusion through the dense iron layer.

Compared to the adjustment of the gas flow and reduction temperature, the coating treatment with additives, typically MgO, CaO, Al_2_O_3_ and SiO_2_, has to take place prior to the reduction step [93,94]. The coating treatment inhibits the sticking in two ways: first, by affecting the morphology and surface properties of the particles, and second, by acting as a physical barrier reducing the frequency of contact of metallic iron surfaces. At this step, the mixing and adsorption, either physically or chemically, have to take place under the restriction of low gas turbulences. Otherwise, the gas flow will entrain the light and fine additive powder. The additives will remain in the product and influence the subsequent steelmaking process [65,93,94,95,96,97,98].

## 5. Conclusions

The need for new ironmaking technologies to reduce CO_2_ emissions in steel production pushes us towards the idea of a hydrogen-based fluidized bed direct reduction process using iron ore ultra-fines directly. Theoretical considerations of the fluid dynamics of iron ore ultra-fines and their usability for hydrogen-based fluidized bed direct reduction are summarized and discussed. The influence of the reduction process, especially the agglomeration phenomenon sticking, are reviewed and evaluated regarding a practical application. The following conclusions can be drawn:

- The behavior of iron ore ultra-fines in a fluidized bed can be classified using the Geldart and Goossens classifications;
- For the fluidization of iron ore-ultra-fines, only the laminar flow, and thus the viscous term, is of interest. The drag coefficient is described sufficiently accurately with 24/Re;
- Changing process conditions, such as temperature, gas properties and gas flow, affect the fluidization as a joint influence of changing fluid dynamics and sticking;
- Sticking leads to an increase in particle size and therefore significantly affects the fluidization conditions and the minimum fluidization velocity;
- The main reason for sticking during hydrogen-based reduction of iron ore ultra-fines is the so-called type 2 sticking—the bonding effect of generated new metallic iron. Type 2 sticking leads to increased adhesion and friction among particles due to the high surface energy and viscosity of the highly active new metallic iron;
- Practical methods to avoid sticking are lowering the reduction temperature and increasing the gas flow rate, hence controlling the iron morphology and the regime of fluidization.

## Figures and Tables

**Table 1:** Annual global iron ore, pig iron and steel production figures for 2016 to 2020 [4,11,12].

Year	2016	2017	2018	2019	2020
Global production figures (M tons)					
Iron ore	2116	2163	2316	2336	-
Pig iron/Hot metal	1174	1186	1253	1282	1319
DRI/HBI	78	92	107	111	106
Crude steel	1633	1736	1825	1875	1878
Share of crude steel production (%)					
BOF	74.0	71.6	70.8	71.6	73.2
EAF	25.5	28.0	28.7	27.9	26.3
Others	0.5	0.5	0.5	0.5	0.5

## Data Availability

Data sharing not applicable. No new data were created or analyzed in this study.
